# Supplementary material for: scReadSim: a single-cell RNA-seq and ATAC-seq read simulator
Source: Nat Commun. 2023 Nov 18;14:7482. doi: 10.1038/s41467-023-43162-w (PMC10657386; doi:10.1038/s41467-023-43162-w)
Supplement: Supplementary file 1 — Supplementary Information [file 41467_2023_43162_MOESM1_ESM.pdf]

Supplementary Note

To run scReadSim, the following dependent software packages are needed. All of the software packages needed to run scReadSim are freely available.

- MACS3: Version 3.0.0a7 (`https://github.com/macs3-project/MACS`).
- samtools: Version 1.12 (with htslib 1.12) (`http://www.htslib.org/`).
- bedtools: Version 2.29.1 (`https://bedtools.readthedocs.io/en/latest/`).
- seqtk: Version 1.3-r117-dirty (`https://github.com/lh3/seqtk`).
- bowtie2: Version 2.3.4.1 (`http://bowtie-bio.sourceforge.net/bowtie2/index.shtml`).
- fgbio: Version 2.0.1 (`http://fulcrumgenomics.github.io/fgbio/`).
- Seurat: R package Seurat Version 4.0.6.
- scDblFinder: R package scDblFinder Version 1.14.0.
- scDesign2: R package scDesign2 Version 1.0.0.
- scDesign3: R package scDesign3 Version 0.99.5.

In addition, the following software packages were used for the analyses in this work.

Existing read simulators

- minnow: Version 0.1.0 (`https://github.com/COMBINE-lab/minnow`)
- SCAN-ATAC-Sim: `http://scan-atac-sim.gersteinlab.org/`

Dimension reduction methods

- UMAP: Function `umap()` from R package umap Version 0.2.7.0.
- PCA: Function `irlba()` from R package irlba Version 2.3.5.

UMI deduplication tools

- UMI-tools: Version 1.1.2 (`https://github.com/CGATOxford/UMI-tools`).
- STARsolo: Version 2.7.9a (`https://github.com/alexdobin/STAR`).

- Alevin: Software Alevin (integrated into the software salmon Version 1.8.0) (https://salmon.readthedocs.io/en/latest/alevin.html).
- cellranger: Version 7.0.0 (https://support.10xgenomics.com/single-cell-gene-expression/software/pipelines/latest/what-is-cell-ranger).

Peak-calling tools

- MACS3: Version 3.0.0a7 (https://github.com/macs3-project/MACS).
- SEACR: Version 1.3 (https://github.com/FredHutch/SEACR).
- HOMER: Software HOMER Version 4.11 (http://homer.ucsd.edu/homer/index.html).
- HMMRATAC: Version 1.2.10 (https://github.com/LiuLabUB/HMMRATAC).

Other tools

- Jellyfish: Version 2.3.0 (https://github.com/gmarcais/Jellyfish).
- Intervene: Python package Intervene Version 0.41.0 (https://intervene.readthedocs.io/en/latest/install.html).

Supplementary Figures

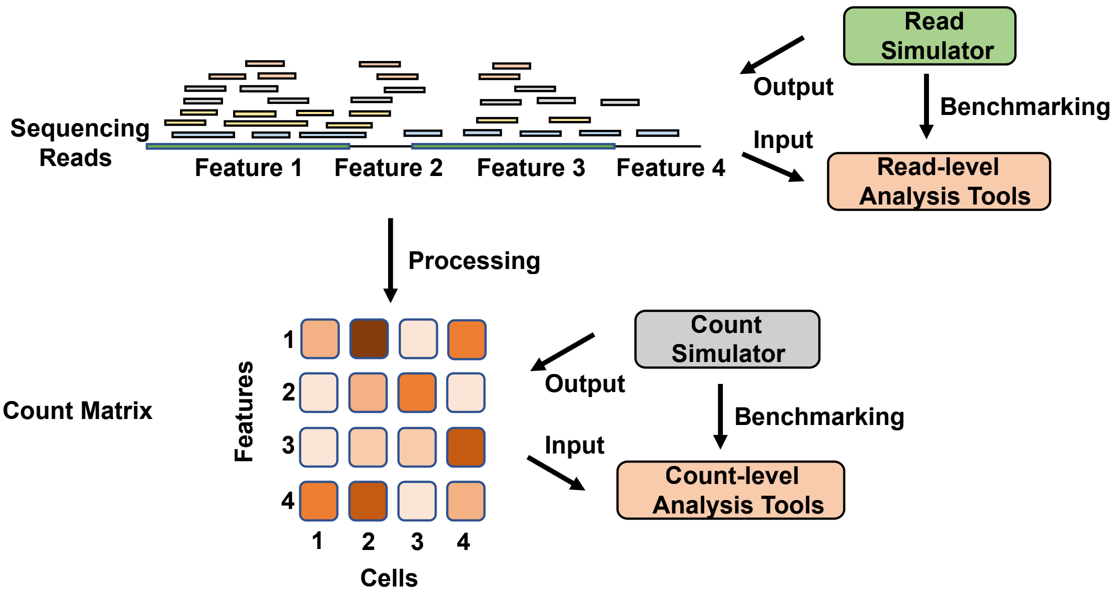

Supplementary Fig. 1: Relationships between single-cell sequencing data analysis tools and simulators. Read-level analysis tools process sequencing reads directly, while count-level analysis tools input a processed sequencing read count matrix. To benchmark count-level analysis tools, researchers need count simulators that generate synthetic sequencing read counts. However, count simulators cannot provide ground truths for benchmarking read-level analysis tools.

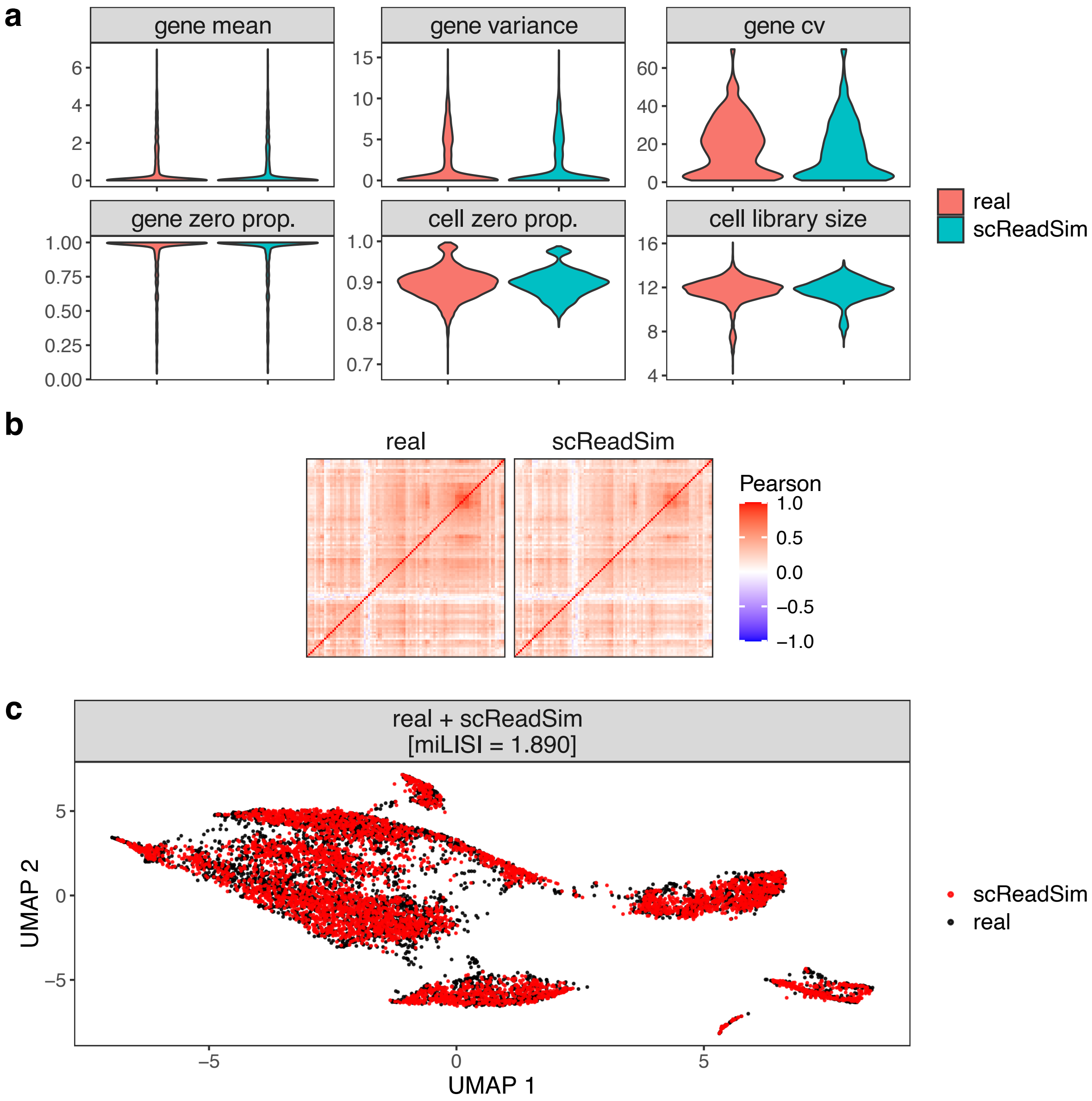

Supplementary Fig. 2: scReadSim's synthetic data resemble real mouse 10x single-cell Multiome dataset (the RNA-seq modality only) [1] at the UMI-count level. **a**, Summary statistics of the UMI count matrix at the gene level (mean, variance, coefficient of variance (cv), and zero proportion) and the cell level (zero proportion and cell library size). **b**, Correlations among the 100 top-expressed genes in the synthetic and real count matrices. The top-expressed genes are defined based on the real count matrix. **c**, UMAP visualization of the pooled real and synthetic cells. miLISI measures the similarity of real and synthetic cells in the UMAP space: the miLISI value ranges between 1 and 2, with 2 indicating a perfect mixing of real and synthetic cells.

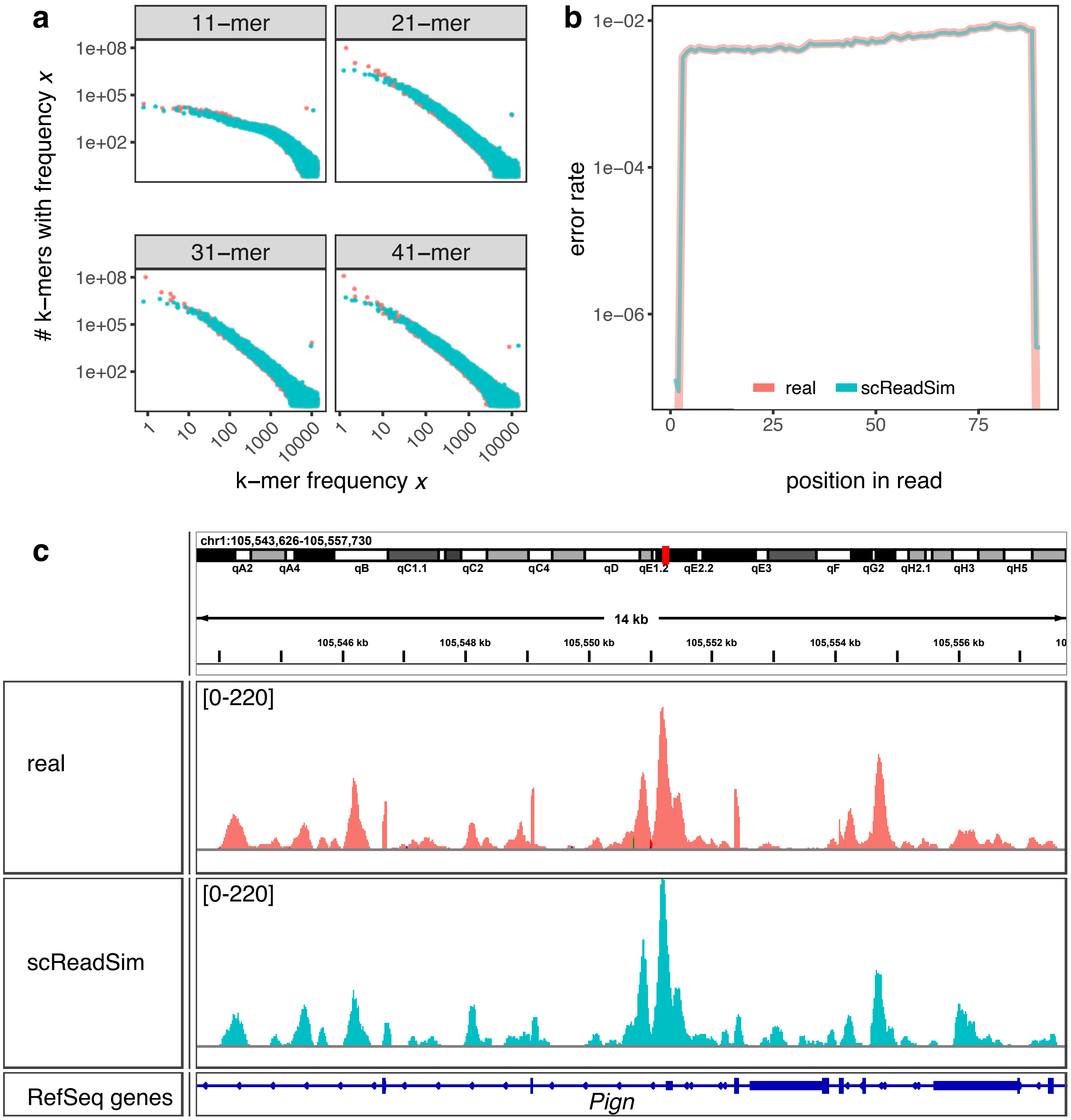

Supplementary Fig. 3: scReadSim's synthetic data resemble real mouse 10x single-cell Multiome dataset (the RNA-seq modality only) [1] at the read-sequence level. a, Comparison of the k-mer spectra between scReadSim's synthetic data and real data. The x-axis refers to the occurrence frequency of a specific k-mer, and the y-axis represents the number of unique k-mers with this frequency. Both the x-axis and y-axis are on the $\log_{10}$ scale. **b**, Comparison of the error rate per base call within a read between scReadSim's synthetic data and real data. The x-axis represents the positions of bases within a read, and the y-axis refers to the substitution error rate in each position. **c**, Read coverage comparison of the real and synthetic BAM files in the IGV genome browser [2]. The genome browser's track height is set to 220 for both tracks.

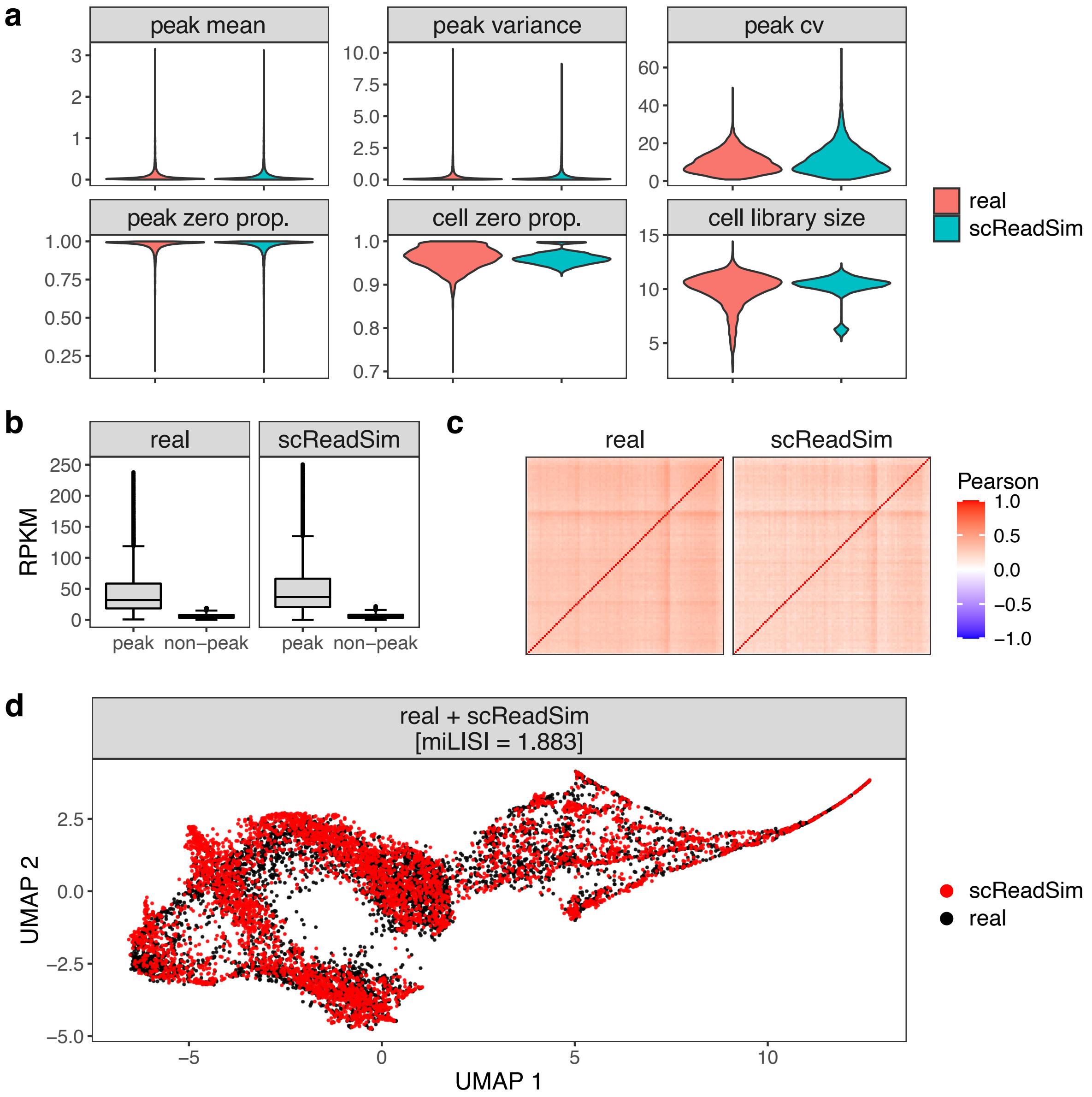

Supplementary Fig. 4: scReadSim's synthetic data resemble real mouse 10x single-cell Multiome dataset (the ATAC-seq modality only) [1] at the read-count level. **a**, Summary statistics of the count matrix at the peak level (mean, variance, coefficient of variance (cv), and zero proportion) and the cell level (zero proportion and cell library size). **b**, Comparison of the RPKM (Reads Per Kilobase Million) value distributions in peak and non-peak regions between scReadSim's synthetic data and real data. Peaks ($n_{\text{peak}} = 13606$) and non-peaks ($n_{\text{non-peak}} = 24286$) are obtained using MACS3 from the real BAM file (Methods). **c**, Correlations among the 100 top-open peaks in the synthetic and real count matrices. The top-open peaks are defined based on the real count matrix. **d**, UMAP visualization of the pooled real and synthetic cells. miLISI measures the similarity of real and synthetic cells in the UMAP space: the miLISI value ranges between 1 and 2, with 2 indicating a perfect mixing of real and synthetic cells.

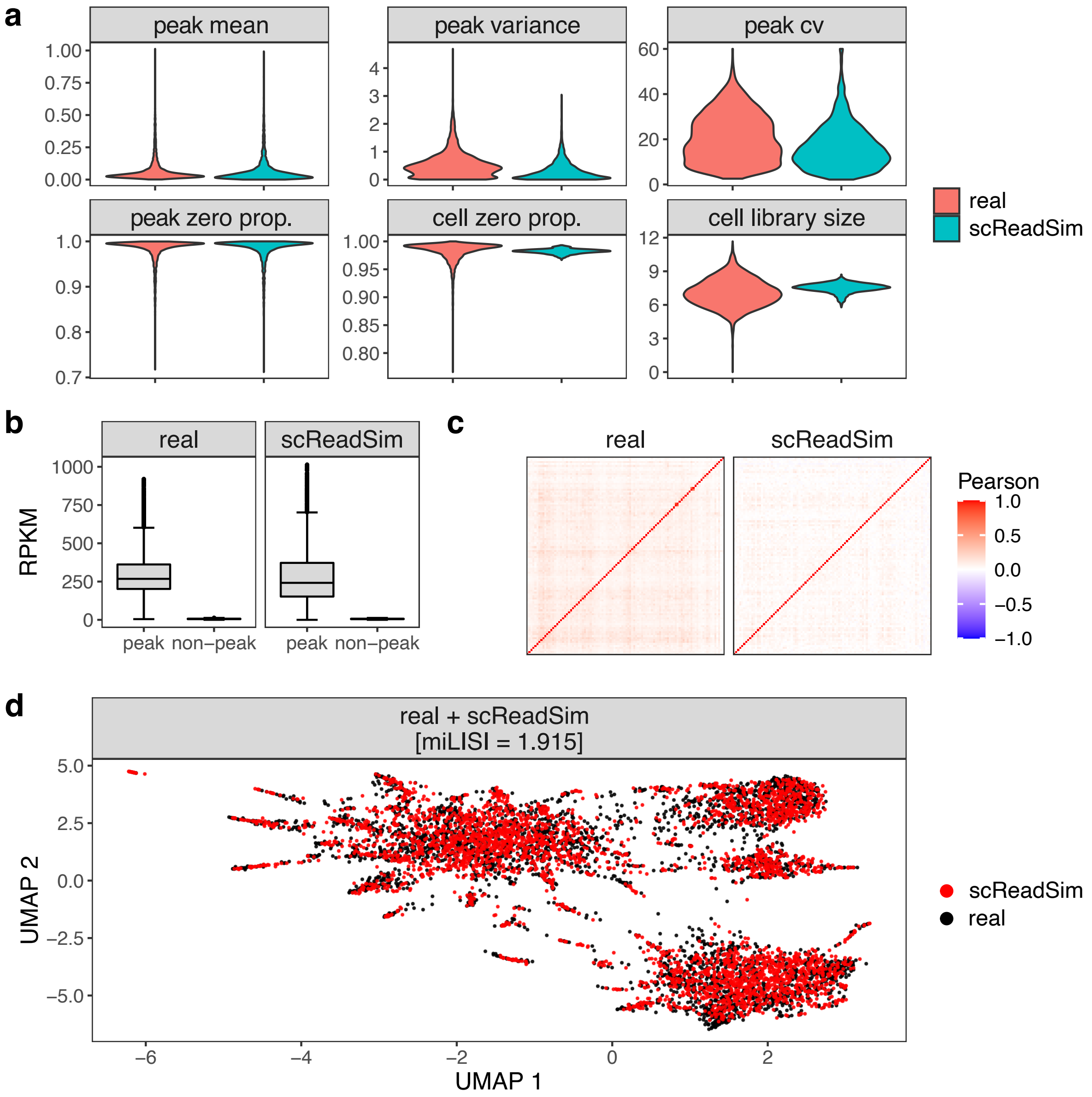

Supplementary Fig. 5: scReadSim's synthetic data resemble real sci-ATAC-seq data [3] at the read-count level. **a**, Summary statistics of the count matrix at the peak level (mean, variance, coefficient of variance (cv), and zero proportion) and the cell level (zero proportion and cell library size). **b**, Comparison of the RPKM (Reads Per Kilobase Million) value distributions in peak and non-peak regions between scReadSim's synthetic data and real data. Peaks ($n_{\text{peak}} = 3644$) and non-peaks ($n_{\text{non-peak}} = 12816$) are obtained using MACS3 from the real BAM file (Methods). **c**, Correlations among the 100 top-open peaks in the synthetic and real count matrices. The top-open peaks are defined based on the real count matrix. **d**, UMAP visualization of the pooled real and synthetic cells. miLISI measures the similarity of real and synthetic cells in the UMAP space: the miLISI value ranges between 1 and 2, with 2 indicating a perfect mixing of real and synthetic cells.

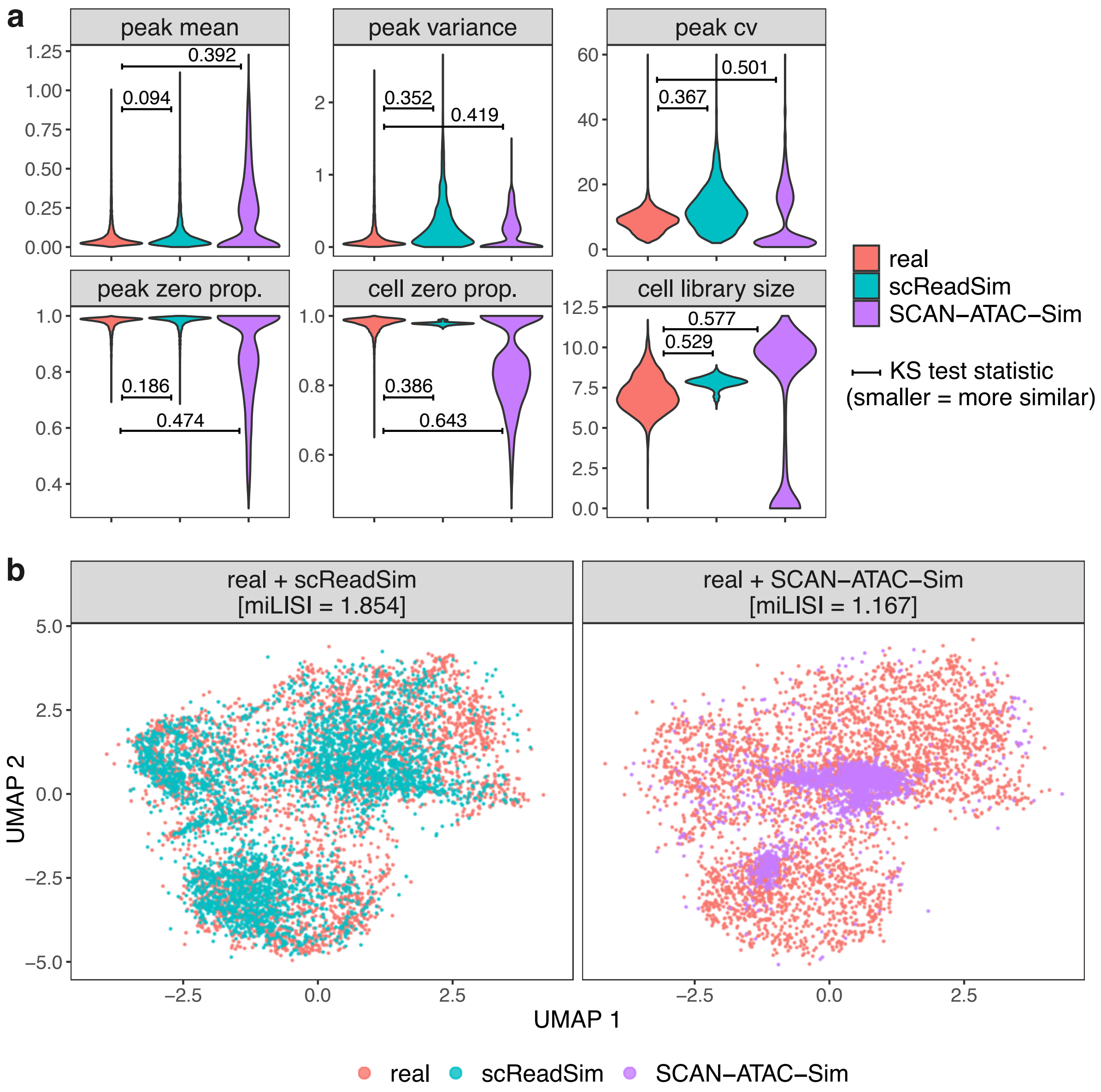

Supplementary Fig. 6: Synthetic cells generated by SCAN-ATAC-Sim do not mimic real cells at the read-count level. **a**, Summary statistics of the count matrix at the peak level (mean, variance, coefficient of variance (cv), and zero proportion) and the cell level (zero proportion and cell library size). Kolmogorov–Smirnov tests (KS tests) are performed to compare the empirical distributions of summary statistics between the real and synthetic read count matrices. Smaller KS statistics indicate that the synthetic data mimic the real data better. **b**, UMAP visualization of the pooled real and synthetic cells. miLISI measures the similarity of real and synthetic cells in the UMAP space: the miLISI value ranges between 1 and 2, with 2 indicating a perfect mixing of real and synthetic cells.

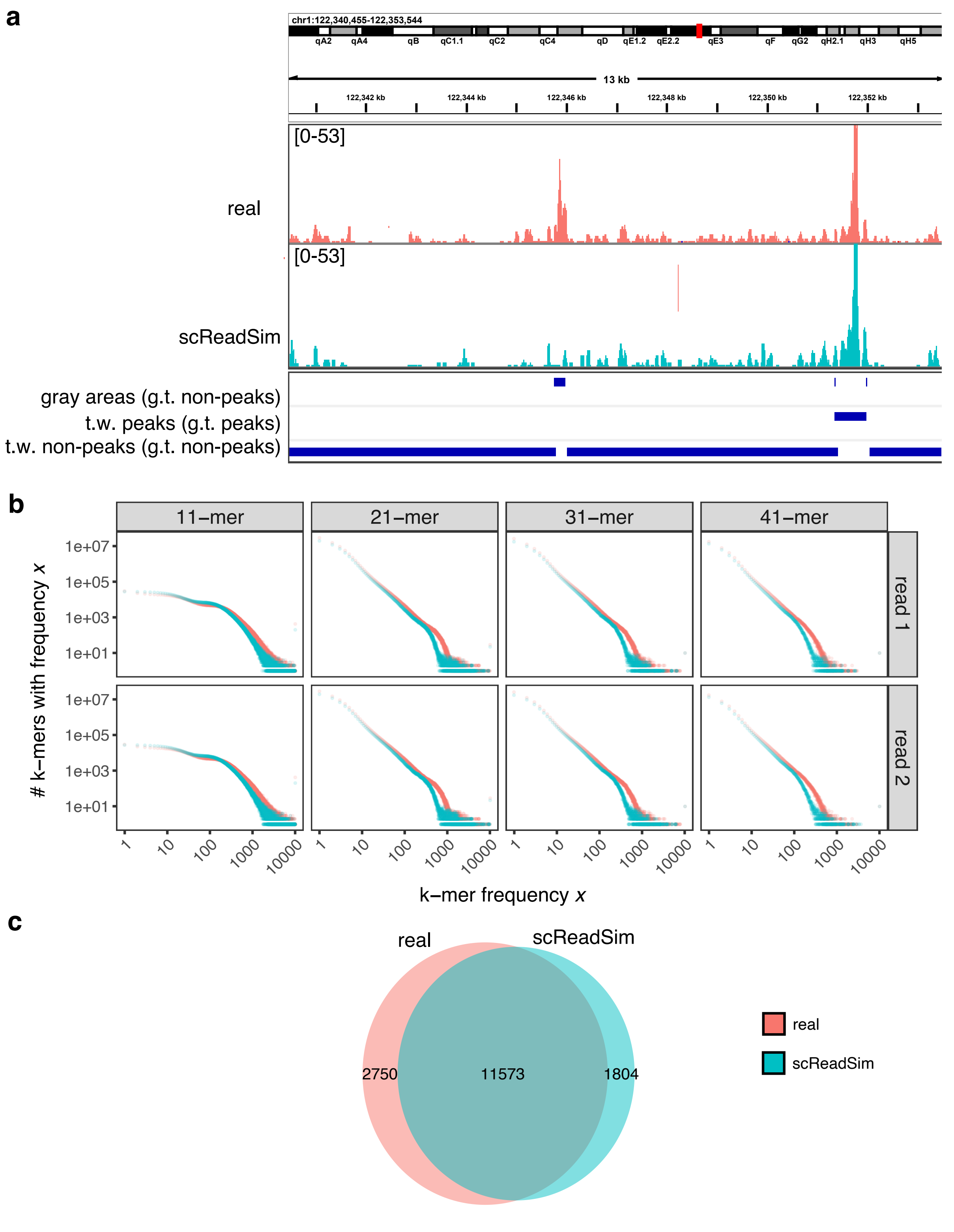

Supplementary Fig. 7: scReadSim's synthetic data resemble real mouse 10x single-cell Multiome dataset (the ATAC-seq modality only) [1] at the read-sequence level. **a**, Read coverage comparison of the real and synthetic BAM files in the IGV genome browser [2]. The genome browser's track height is set to 53 for both tracks. Gray areas, trustworthy (t.w.) peaks, and trustworthy non-peaks are user-specified or scReadSim-identified from real data. scReadSim converts gray areas to ground-truth (g.t.) non-peaks, and it maintains trustworthy peaks and non-peaks as ground-truth peaks and non-peaks, respectively, in synthetic data. **b**, Comparison of the k-mer spectra between scReadSim's synthetic data and real data. The x-axis refers to the occurrence frequency of a specific k-mer, and the y-axis represents the number of unique k-mers with this frequency. Both the x-axis and y-axis are on the $\log_{10}$ scale. **c**, A Venn diagram of MACS3's called peaks from scReadSim's synthetic data and real data. A peak called from real data (referred to as a "real peak") is considered to overlap with a peak called from synthetic data (referred to as a "synthetic peak") if the real peak has at least 101 bp (half of the shortest real peak's length) overlapped by the synthetic peak.

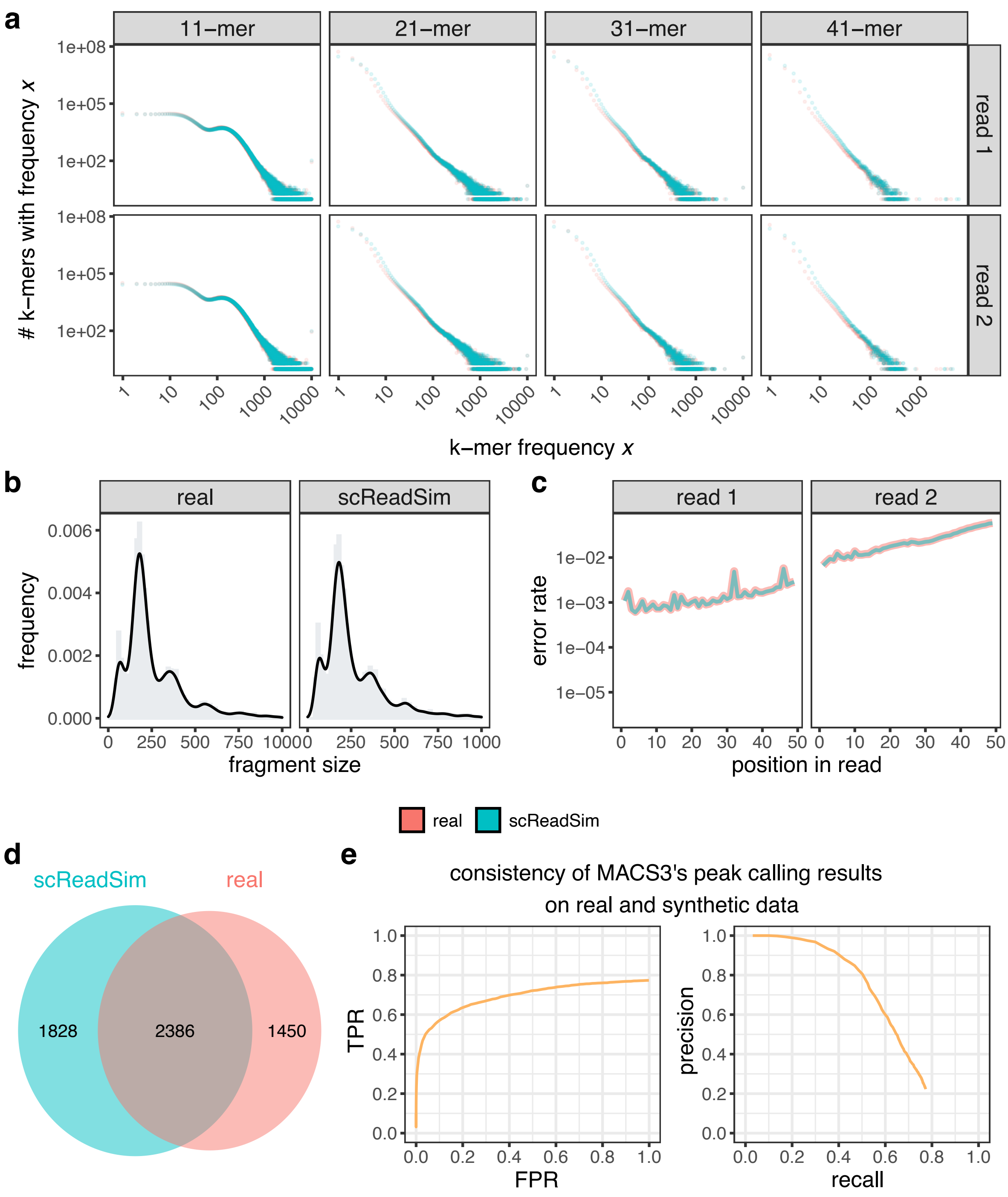

Supplementary Fig. 8: scReadSim's synthetic data resemble real sci-ATAC-seq data [3] at the read-sequence level. **a**, Comparison of the k-mer spectra between scReadSim's synthetic data and real data. The x-axis refers to the occurrence frequency of a specific k-mer, and the y-axis represents the number of unique k-mers with this frequency. Both the x-axis and y-axis are on the $\log_{10}$ scale. **b**, Comparison of the fragment-size distributions between scReadSim's synthetic data and real data. **c**, Comparison of the error rate per base call within a read between scReadSim's synthetic data and real data. The x-axis represents the positions of bases within a read, and the y-axis refers to the substitution error rate in each position. **d**, A Venn diagram of MACS3's called peaks from scReadSim's synthetic data and real data. A peak called from real data (referred to as a "real peak") is considered to overlap with a peak called from synthetic data (referred to as a "synthetic peak") if the real peak has at least 135 bp (half of the shortest real peak's length) overlapped by the synthetic peak. **e**, True positive rate (TPR) vs. false positive rate (FPR) (left) and precision vs. recall (right) for evaluating the synthetic peaks in (d) by treating the real peaks in (d) as truths. The TPR, equivalent to the recall, is the proportion of real peaks that have at least 135 bp overlapped by any synthetic peak; the FPR is the proportion of real non-peaks (the regions complementary to the real peaks) that have at least 135 bp overlapped by any synthetic peak; the precision is the proportion of synthetic peaks that overlap at least 135 bp of any real peak.

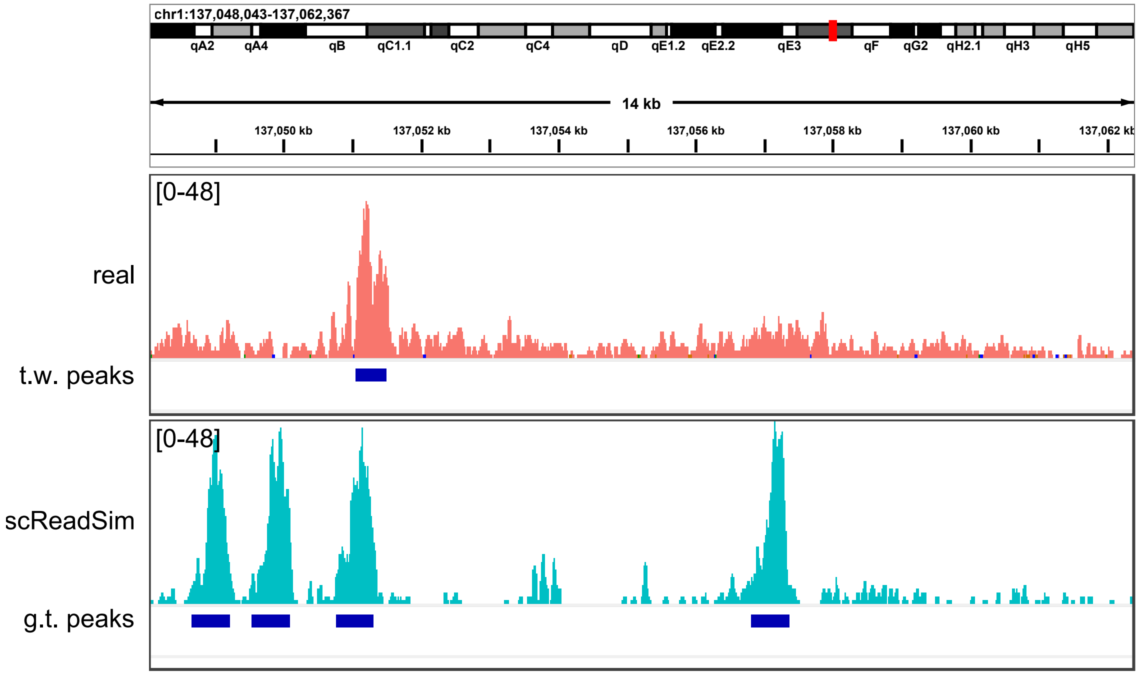

Supplementary Fig. 9: IGV genome browser [2] visualization of scReadSim's synthetic sci-ATAC-seq reads with user-designed ground-truth (g.t.) peaks (bottom track). From the real sci-ATAC-seq reads (top track) [3], the trustworhy (t.w.) peaks are called by MACS3 and used to train scReadSim. The genome browser's track height is set to 48 for both tracks.

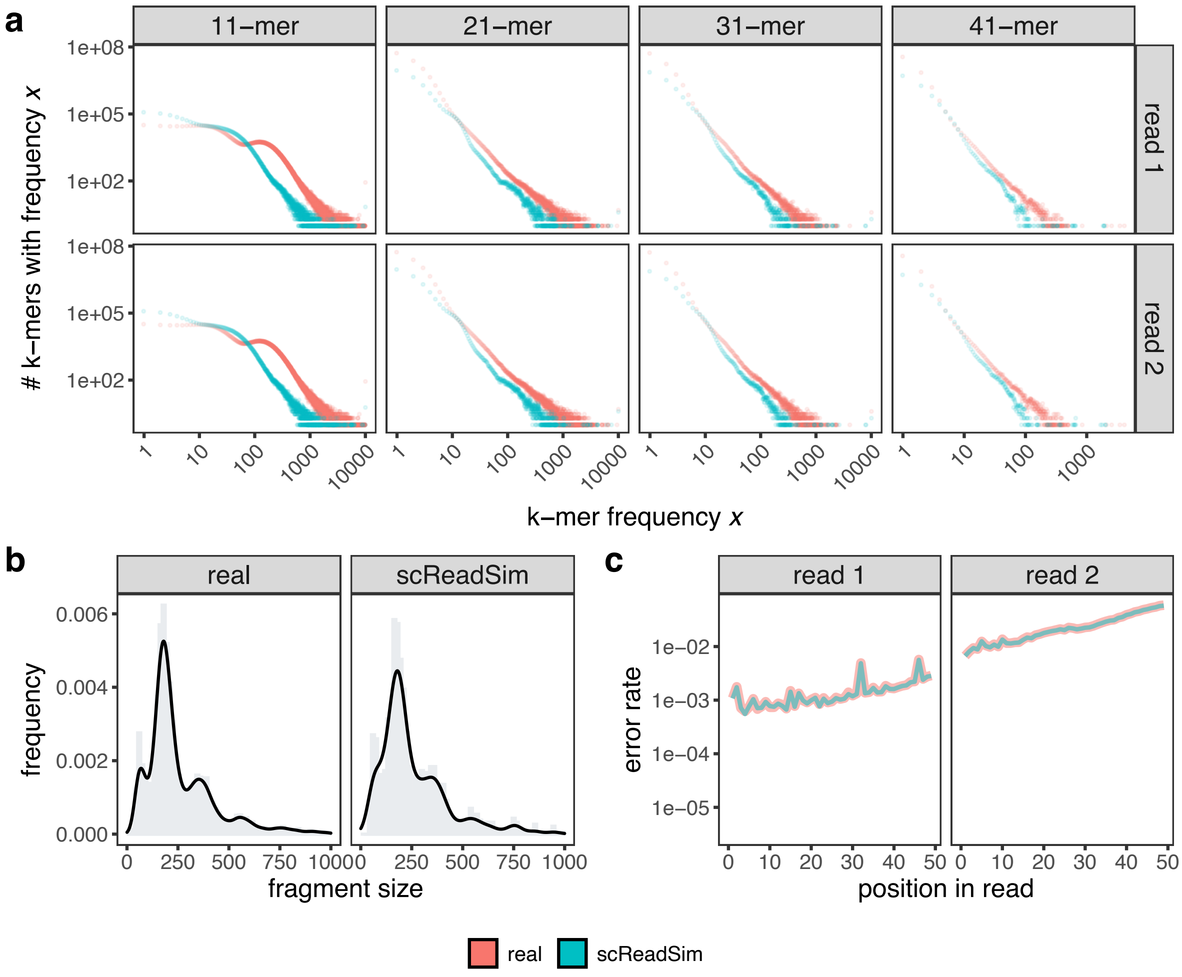

Supplementary Fig. 10: scReadSim's synthetic data with user-designed open chromatin regions resemble real sci-ATAC-seq data [3] at the read-sequence level. **a**, Comparison of the k-mer spectra between scReadSim's synthetic data and real data. The x-axis refers to the occurrence frequency of a specific k-mer, and the y-axis represents the number of unique k-mers with this frequency. Both the x-axis and y-axis are on the $\log_{10}$ scale. The real reads and scReadSim's synthetic reads are expected to have different k-mer spectra because they correspond to different open chromatin regions. **b**, Comparison of the fragment-size distributions between scReadSim's synthetic data and real data. **c**, Comparison of the error rate per base call within a read between scReadSim's synthetic data and real data. The line plot's x-axis represents the positions of bases within a read, and the y-axis refers to the substitution error rate corresponding to each position.

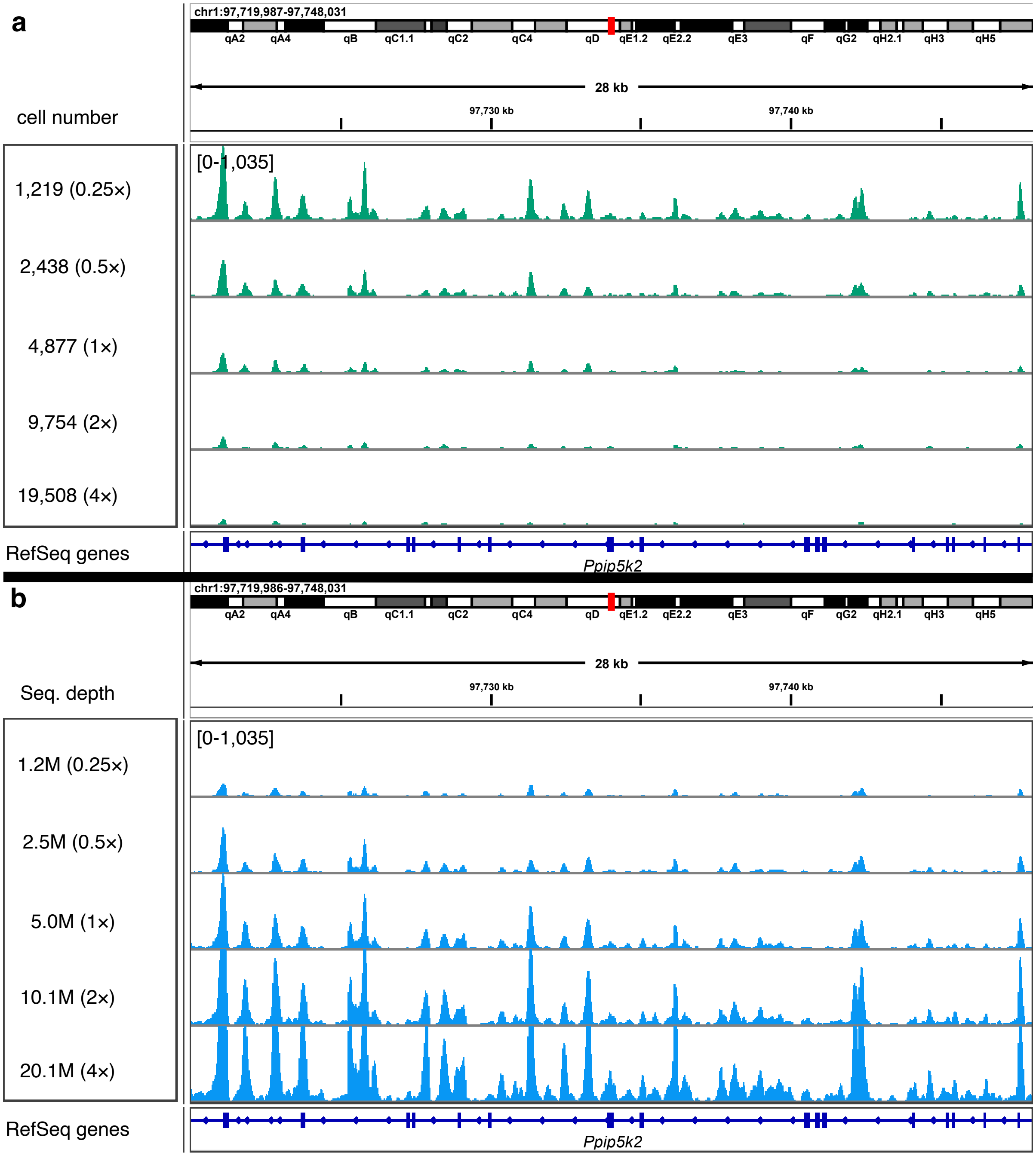

Supplementary Fig. 11: scReadSim allows varying cell number and sequencing depth as input parameters, with the corresponding synthetic data visualized in the IGV genome browser [2]. **a**, Varying numbers of synthetic cells. The track names indicate the multiplication factors: '$1\times$' specifies the cell number in the real data; '$4\times$' specifies 4 times the real cell number. All five synthetic datasets are downsampled to $1{,}219$ cells for a fair comparison. **b**, Varying sequencing depths in synthetic data. The track names indicate the multiplication factors: '$1\times$' specifies the sequencing depth in the real data; '$4\times$' specifies 4 times the real sequencing depth. For better visualization, the genome browser's track height is set to $1{,}035$ for all 10 tracks.

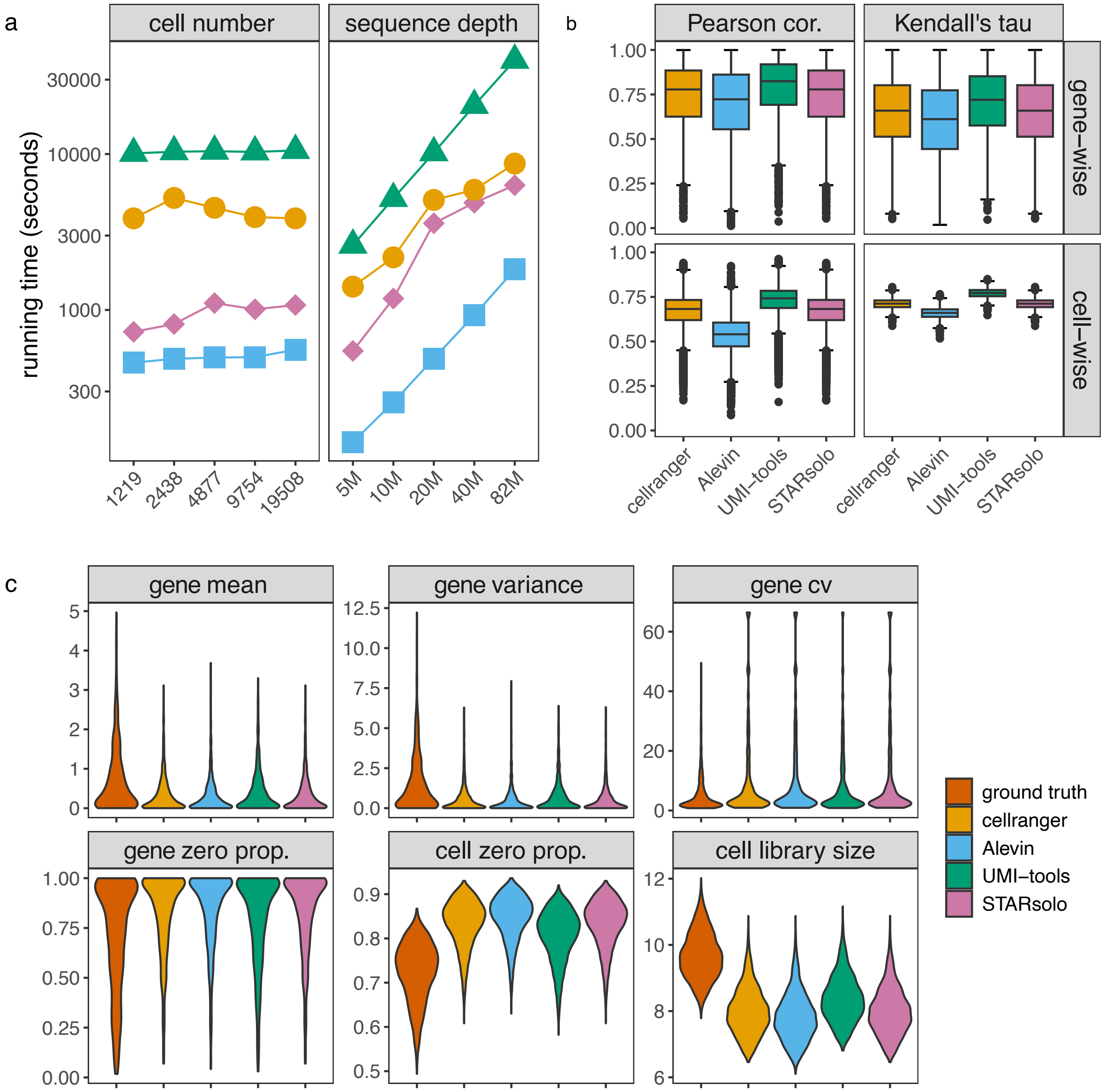

Supplementary Fig. 12: Benchmark of UMI deduplication tools using scReadSim's synthetic scRNA-seq reads. The ground-truth UMI count matrix is the gene-by-cell UMI count matrix generated by scReadSim. Four deduplication tools are considered: cellranger, Alevin, UMI-tools, and STARsolo. **a**, Time usage of deduplication tools on synthetic datasets with varying cell numbers or sequencing depths. The y-axis indicates the time lapse (in seconds), and the x-axis reflects the number of synthetic cells or the total number of synthetic reads (sequencing depth). **b**, Cell-wise ($n_{\text{cell}} = 3256$) and gene-wise ($n_{\text{gene}} = 17652$) correlations (Pearson correlation and Kendell's tau) between the ground-truth UMI count matrix and each deduplication tool's output UMI count matrix. **c**, Summary statistics of the UMI count matrices at the gene level (mean, variance, coefficient of variance (cv), and zero proportion) and the cell level (zero proportion and cell library size).

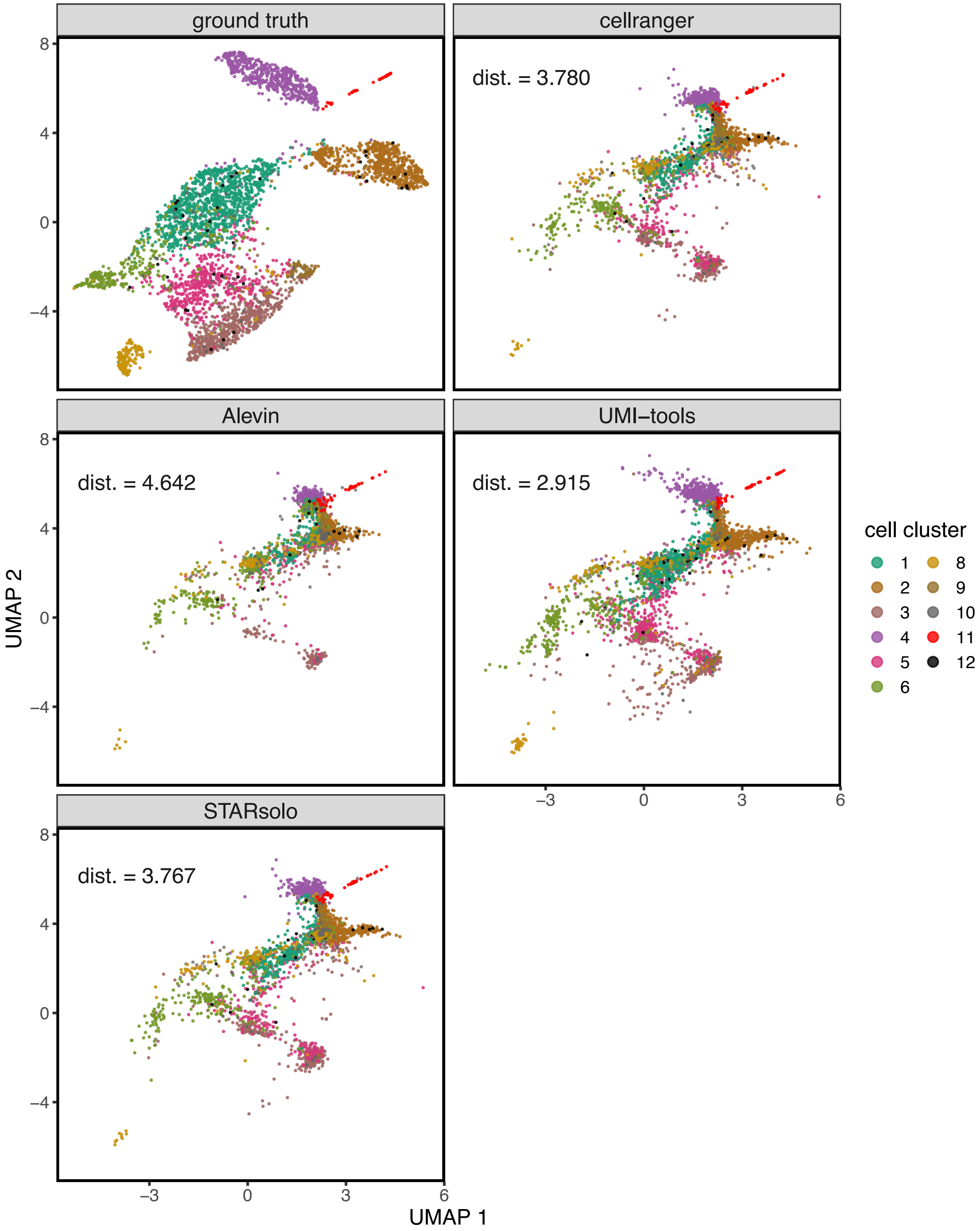

Supplementary Fig. 13: UMAP visualizations of the ground-truth UMI count matrix and each deduplication tool's output UMI count matrix. Cells are colored by the cell clusters outputted by scReadSim (the clusters are from the real data used to train scReadSim). The synthetic cells' deduplicated UMI counts are projected to the UMAP space defined by the same cells' ground-truth UMI counts. We computed the Euclidean distance between each synthetic cell's two UMAP coordinates, calculated based on the cell's ground-truth UMI counts and deduplicated UMI counts, respectively. The mean value of the Euclidean distances of all synthetic cells is displayed for each UMI deduplication tool: a smaller value indicates that the deduplicated UMI count matrix better agrees with the ground truth UMI count matrix in UMAP visualization.

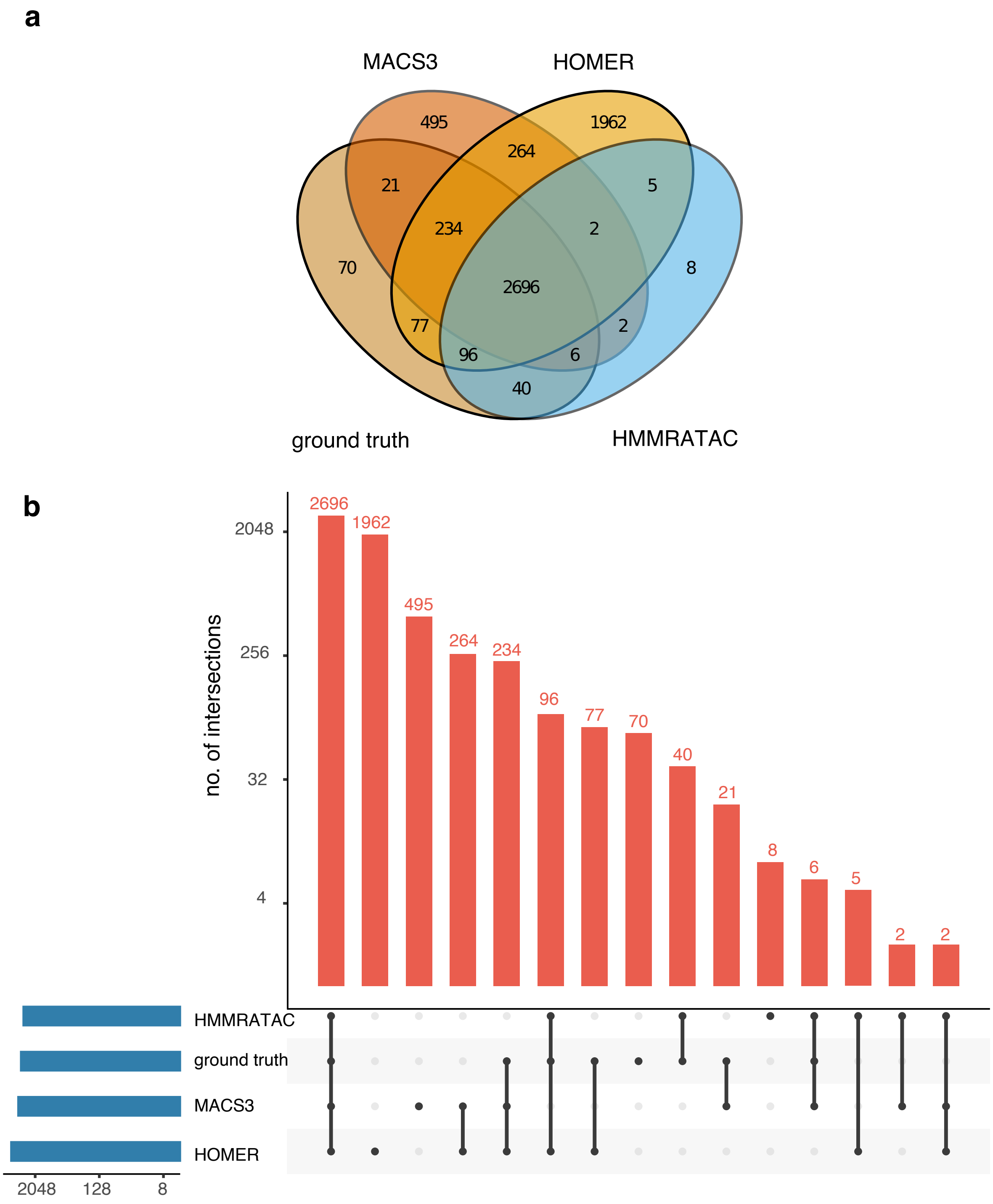

Supplementary Fig. 14: Benchmark of peak-calling tools (MACS3, HOMER, and HMMRATAC) using scReadSim's synthetic scATAC-seq reads generated from user-designed ground-truth peaks (i.e., open chromatin regions) (Methods). The Venn diagram (**a**) and upset plot (**b**) of the user-designed open chromatin regions and the peaks called by MACS3, HOMER, and HMMRATAC. See Supplementary Figure 15 for the comparison results that include the peak-calling tool SEACR, whose called peaks differ drastically from the ground truth and the peaks called by MACS, HOMER, and HMMRATAC.

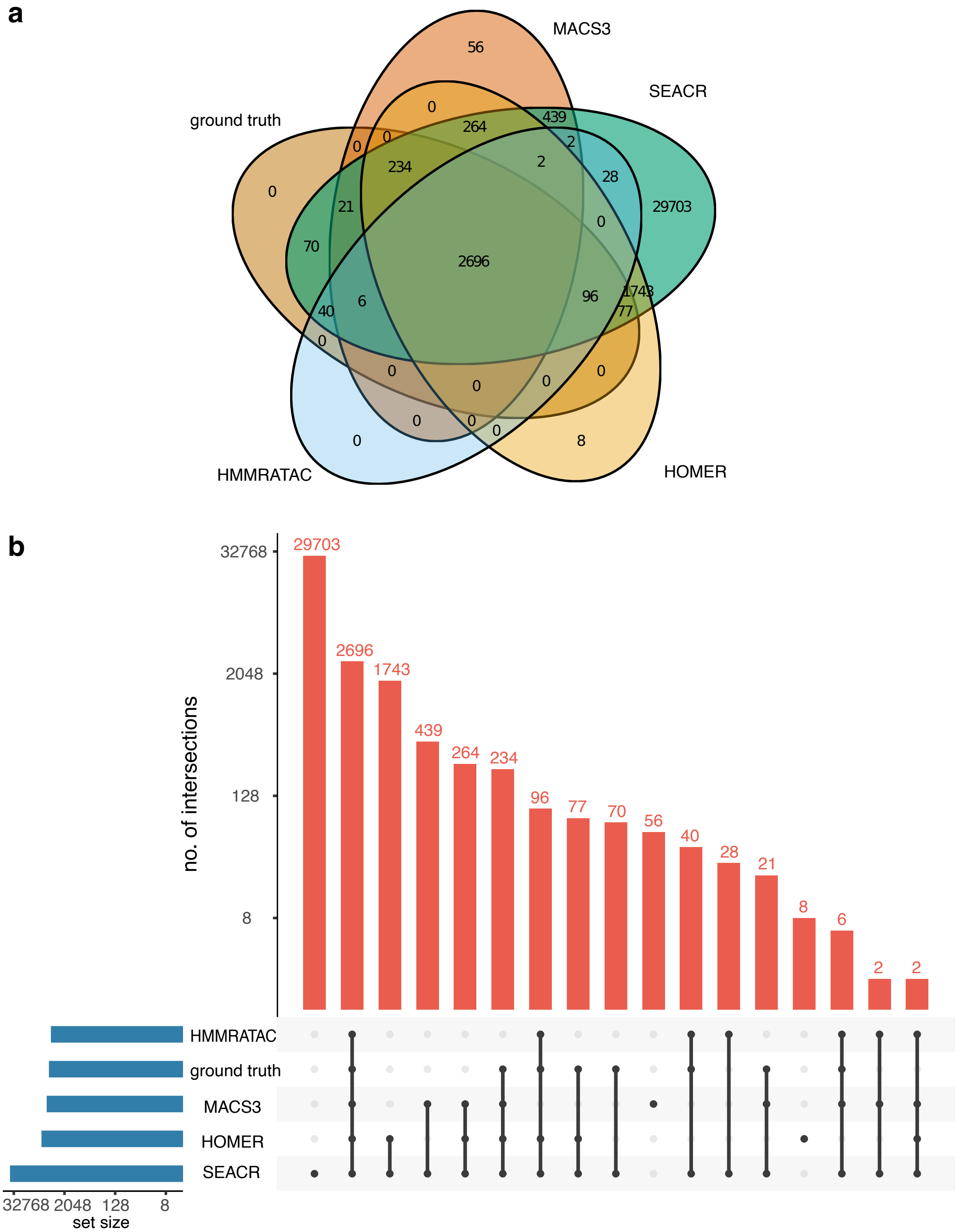

Supplementary Fig. 15: Benchmark of peak-calling tools (MACS3, SEACR, HOMER, and HMMRATAC) using scReadSim's synthetic scATAC-seq reads generated from user-designed ground-truth peaks (i.e., open chromatin regions). The Venn diagram (**a**) and upset plot (**b**) of the user-designed open chromatin regions and the peaks called by MACS3, SEACR, HOMER, and HMMRATAC.

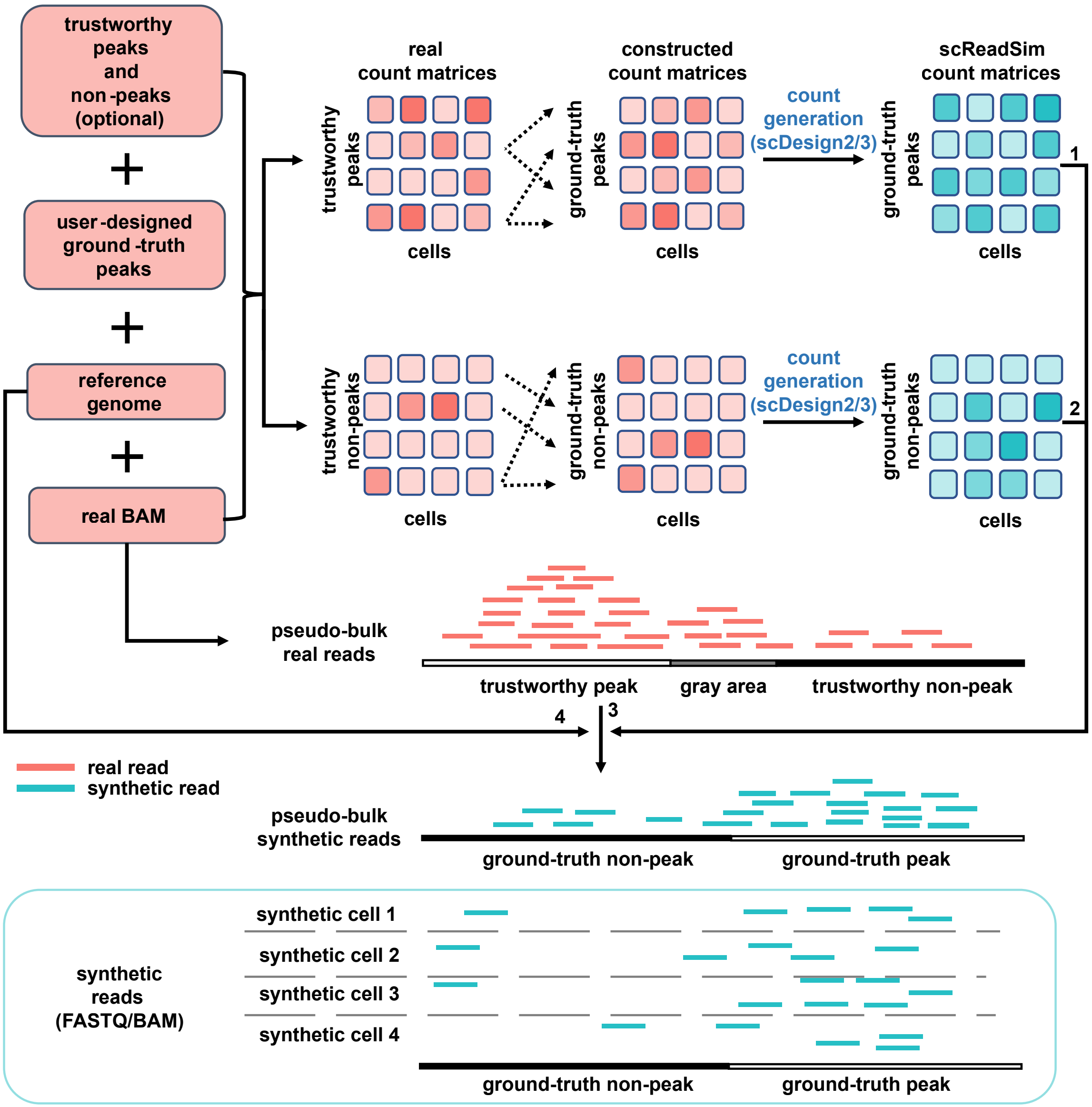

Supplementary Fig. 16: Workflow of scReadSim for generating scATAC-seq reads with user-designed open chromatin regions (i.e., ground-truth peaks). The input includes a BAM file, the corresponding reference genome, trustworthy peak and non-peak lists, and a ground-truth peak list that the synthetic scATAC-seq reads will be generated accordingly. Specifically, if users do not input trustworthy peaks and non-peaks, scReadSim provides two options; see the subsection "scReadSim for scATAC-seq" for detail. Given ground-truth peaks, scReadSim defines the inter-peaks as the ground-truth non-peaks. Based on trustworthy peaks and non-peaks, scReadSim summarizes scATAC-seq reads in the input BAM file into a trustworthy-peak-by-cell count matrix and a trustworthy-non-peak-by-cell count matrix. Next, scReadSim defines a mapping between trustworthy and ground-truth peaks and constructs a ground-truth-peak-by-cell count matrix by extracting the corresponding trustworthy-peak-by-cell count matrix's entries. Similarly, scReadSim constructs a ground-truth-non-peak-by-cell count matrix from the trustworthy-non-peak-by-cell count matrix. Further, scReadSim trains the count simulator scDesign2 [4] (if the cells belong to distinct clusters; otherwise, scDesign3 [5] can be used if the cells follow continuous trajectories) on the ground-truth-peak-by-cell and ground-truth-non-peak-by-cell count matrices to generate the corresponding synthetic count matrices for the ground-truth peaks and non-peaks. Last, scReadSim generates synthetic reads based on the synthetic count matrices (Input 1 and 2), the input BAM file (Input 3), and the reference genome (Input 4). The synthetic reads are outputted in the FASTQ or BAM format.

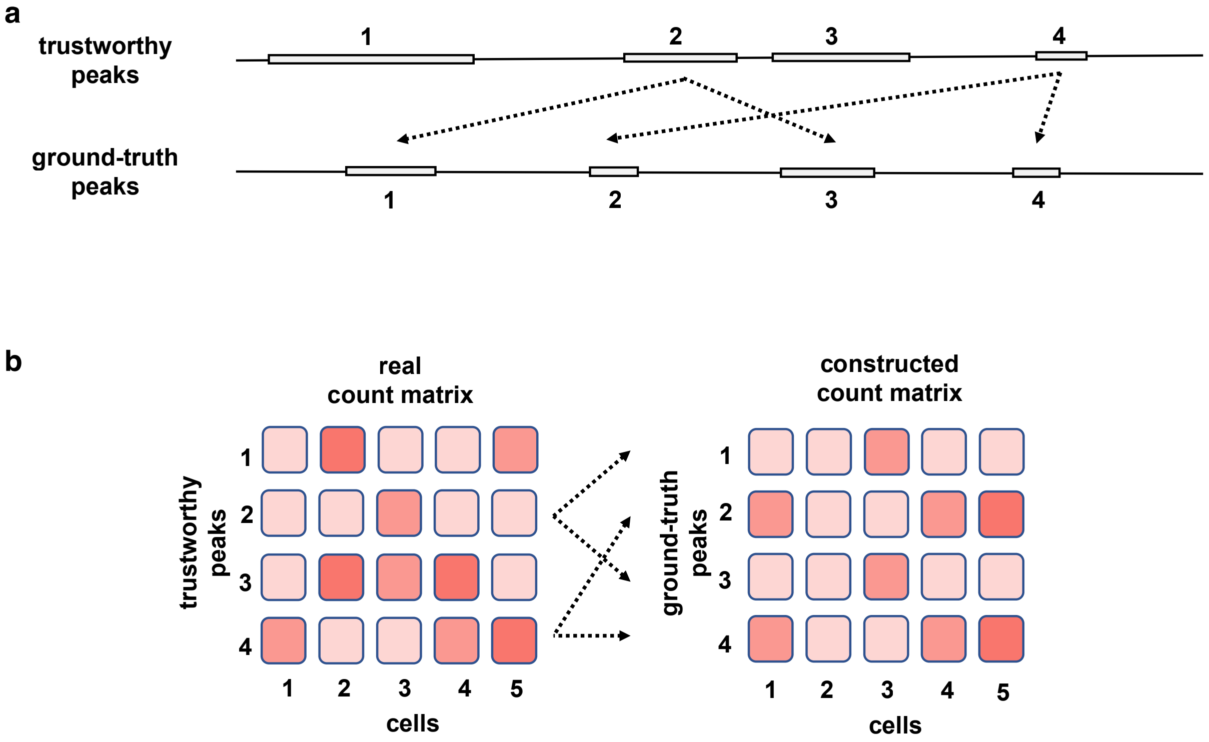

Supplementary Fig. 17: The peak-mapping step in scReadSim's data generation with user-designed open chromatin regions (i.e., ground-truth peaks). **a**, The mapping between trustworthy peaks and ground-truth peaks. For every ground-truth peak, scReadSim finds its most similar trustworthy peak (in the same chromosome) in terms of length. **b**, The constructed count matrix for ground-truth peaks. For every ground-truth peak, scReadSim adds the corresponding trustworthy peak's row in the trustworthy peak-by-cell count matrix to the ground-truth peak-by-cell count matrix.

Supplementary Tables

Supplementary Table 1: Comparison of scReadSim, minnow, and SCAN-ATAC-Sim

Property \ Tools	scReadSim	minnow	SCAN-ATAC-Sim
Modality	scRNA-seq<br>scATAC-seq	scRNA-seq	scATAC-seq
Input	scRNA-seq reads<br>or scATAC-seq reads<br>gene annotations	UMI count matrix<br>gene annotations	ATAC-seq reads<br>peak lists
Output	FASTQ<br>or BAM	FASTQ	BED
Ground truths	UMI counts<br>open chromatin regions	UMI counts	N/A
Read-sequence preservation[1]	✓	✓✗[2]	✗
Read-count preservation[3]	✓	✓	✗
Read-coverage preservation[4]	✓	✗	✓✗[5]
Whole-genome read simulation	✓	✗	✓

[1]: "Read-sequence preservation" means whether the simulator preserves the characteristics of real read sequences, such as k-mer spectra and sequencing errors.
[2]: Since minnow does not input scRNA-seq reads, it uses its internal, pre-trained model to generate sequencing errors.
[3]: "Read-count preservation" means whether the simulator generates synthetic cells that mimic real cells regarding gene or peak counts.
[4]: "Read-coverage preservation" means whether the simulator's synthetic reads have a coverage profile mimicking that of real reads at the pseudo-bulk level.
[5]: SCAN-ATAC-Sim only inputs pseudo-bulk ATAC-seq reads and thus does not preserve read coverage at the single-cell level.

Supplementary References

[1] 10xGenomics. Fresh embryonic e18 mouse brain (5k), single cell multiome atac + gene expression dataset by cell ranger arc 2.0.0. https://www.10xgenomics.com/resources/datasets/fresh-embryonic-e-18-mouse-brain-5-k-1-standard-2-0-0, 2019.

[2] James T Robinson, Helga Thorvaldsdóttir, Wendy Winckler, Mitchell Guttman, Eric S Lander, Gad Getz, and Jill P Mesirov. Integrative genomics viewer. *Nature biotechnology*, 29(1):24–26, 2011.

[3] Darren A Cusanovich, Andrew J Hill, Delasa Aghamirzaie, Riza M Daza, Hannah A Pliner, Joel B Berletch, Galina N Filippova, Xingfan Huang, Lena Christiansen, William S DeWitt, et al. A single-cell atlas of in vivo mammalian chromatin accessibility. *Cell*, 174(5):1309–1324, 2018.

[4] Tianyi Sun, Dongyuan Song, Wei Vivian Li, and Jingyi Jessica Li. scDesign2: a transparent simulator that generates high-fidelity single-cell gene expression count data with gene correlations captured. *Genome biology*, 22(1):1–37, 2021.

[5] Dongyuan Song, Qingyang Wang, Guanao Yan, Tianyang Liu, Tianyi Sun, and Jingyi Jessica Li. scdesign3 generates realistic in silico data for multimodal single-cell and spatial omics. *Nature biotechnology*, pages 1–6, 2023.
